# The Sex-Related Interplay between TME and Cancer: On the Critical Role of Estrogen, MicroRNAs and Autophagy

**DOI:** 10.3390/cancers13133287

**Published:** 2021-06-30

**Authors:** Paola Matarrese, Gianfranco Mattia, Maria Teresa Pagano, Giada Pontecorvi, Elena Ortona, Walter Malorni, Alessandra Carè

**Affiliations:** 1 Center for Gender Specific Medicine, Istituto Superiore di Sanità, 00161 Rome, Italy; paola.matarrese@iss.it (P.M.); gianfranco.mattia@iss.it (G.M.); mariateresa.pagano@guest.iss.it (M.T.P.); giada.pontecorvi@iss.it (G.P.); elena.ortona@iss.it (E.O.); alessandra.care@iss.it (A.C.); 2 Department of Biology, Faculty of Farmacy, University of Rome Tor Vergata, 00133 Rome, Italy; 3 Center for Global Health, Università Cattolica del Sacro Cuore, 00168 Rome, Italy

**Keywords:** cancer, gender, sex, sex hormones, microRNA, autophagy, tumor microenvironment

## Abstract

**Simple Summary:**

Autophagy is a complex cell process that allow the cell to survive in unfavorable conditions, e.g., in the lack of nutritional elements coming from the environment. Here we focused on the role played by autophagy in the crosstalk between the microenvironment surrounding the tumor and cancer cells. This environment is in fact known as pivotal in determining the growth or the inhibition of a tumor. Cancer progression and response to therapy significantly differ between women and men and the microenvironment, in particular sex hormones and microRNAs, appears a critical factor. Four representative types of cancer, i.e., colon cancer, melanoma, lymphoma, and lung cancer showing sex/gender specificities have been described herein. We underscore that the use of a “gender tailored” approach could provide a better comprehension of the cellular and molecular mechanisms of cancer growth control contributing to the development of novel therapeutic approaches towards an increasingly personalized medicine.

**Abstract:**

The interplay between cancer cells and the tumor microenvironment (TME) has a fundamental role in tumor progression and response to therapy. The plethora of components constituting the TME, such as stroma, fibroblasts, endothelial and immune cells, as well as macromolecules, e.g., hormones and cytokines, and epigenetic factors, such as microRNAs, can modulate the survival or death of cancer cells. Actually, the TME can stimulate the genetically regulated programs that the cell puts in place under stress: apoptosis or, of interest here, autophagy. However, the implication of autophagy in tumor growth appears still undefined. Autophagy mainly represents a cyto-protective mechanism that allows cell survival but, in certain circumstances, also leads to the blocking of cell cycle progression, possibly leading to cell death. Since significant sex/gender differences in the incidence, progression and response to cancer therapy have been widely described in the literature, in this review, we analyzed the roles played by key components of the TME, e.g., estrogen and microRNAs, on autophagy regulation from a sex/gender-based perspective. We focused our attention on four paradigmatic and different forms of cancers—colon cancer, melanoma, lymphoma, and lung cancer—concluding that sex-specific differences may exert a significant impact on TME/cancer interaction and, thus, tumor growth.

## 1. Introduction

### 1.1. Tumor Microenvironment

It has long been known that a tumor mass is constituted by cancer cells and by several other cell types such as smooth muscle cells, endothelial cells, fibroblasts, lymphocytes, macrophages, and, especially in some types of cancer, adipocytes. These cells dynamically interact with the tumor cells and with the components of the extracellular matrix (ECM) to constitute the tumor microenvironment (TME), which significantly contributes to tumor progression [1]. Through humoral factors, such as cytokines, chemokines, hormones, and active metabolites, microRNAs (miRs), and mechanical stimuli, e.g., those mediated by cell adhesion features, TME can exert a critical function by co-stimulating (or co-inhibiting) the clonal selection, thus leading to a metastatic or drug-resistant phenotype as well as contributing to cancer-associated angiogenesis [2]. For instance, tumor-associated macrophages (TAM) and cancer-associated fibroblasts (CAF) can interact with each other and with tumor cells through paracrine mechanisms to produce a tumor-promoting microenvironment. Hence, the TME could be involved in all stages of tumor progression, including epithelial–mesenchymal transition (EMT), migration and invasion, extravasation and colonization at the secondary site of metastasis [3]. Currently, the TME characteristics represent a hallmark of cancer, often with predictive value [4]. The cells embedded in the tumor stroma also play a fundamental role as metabolic support for tumor cells. For example, as a consequence of their metabolic reprogramming induced by cancer cells, the aerobic glycolysis of stromal cells would provide the latter, in addition to ATP, with various metabolites, which are instrumental to the production of further ATP through OXPHOS. In addition, a phagocytic cannibalic behavior exerted by cancer cells could also provide further nutrients to cancer cells [5,6]. This metabolic coupling between stromal and tumor cells would also allow the exchange of metabolites, which are suitable to tumor cells in terms of increasing their proliferation and reducing cell death. This phenomenon is known as the Reverse Warburg Effect [7].

**Figure 1:**
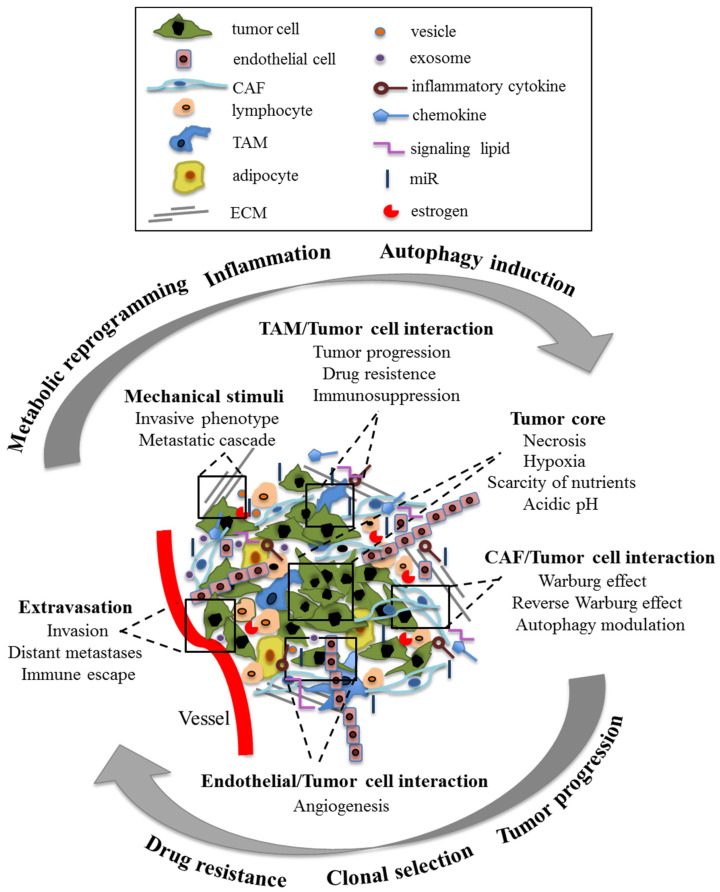
Simplified and exemplary illustration of the main cellular and non-cellular components of the TME that actively participate, through multiple intercellular signals, in tumor progression.

In Figure 1, a simplified scheme of the main cellular and non-cellular components of the TME involved in tumor progression through multiple intercellular signals is reported.

### 1.2. Autophagy

As the TME is often hypoxic and sometimes scarce of nutrients, cancer cells counteract this detrimental issue by activating an elusive survival process, i.e., autophagy, thus adapting to these unfavorable environmental conditions [8]. Autophagy (more precisely the process called macroautophagy) is a homeostatic, highly conserved, genetically driven process by which macromolecules and organelles can be degraded and recycled within the cells. It is a cytoprotective process driven by specific genes: the autophagy genes (*ATGs*) [9]. Thirty-six proteins produced by these genes, the ATG proteins, have been identified so far in yeast, each with a respective orthologue detectable in mammalian cells. In normal non-transformed cells, autophagy represents a protective mechanism that can be increased in the case of lack of nutrients or in the presence of cell-damaging stressors. Notably, it has been suggested that autophagy inhibition in non-transformed cells could contribute to cancerogenesis [10] or that it can play an essential role in promoting tumor cell survival, by also conferring resistance to anticancer drugs [11] and favoring survival of dormant cancer cells [12]. The interplay between TME and autophagy is thus pivotal. For instance, the hypoxic and anoxic conditions of the TME have been shown to induce autophagy via the 5′ adenosine monophosphate-activated protein kinase (AMPK), the PKR-like ER kinase (PERK), and the Hypoxia-Inducible Factor 1-alpha (HIF-1α)/Forkhead box O3 (FOXO3) signaling pathways [13]. Furthermore, inflammation, often associated with tumor progression, can induce autophagy, mainly through the activity of the nuclear factor kappa B (NF-κB), a major player involved in the inflammatory response [14].

Recently, a very selective form of autophagy, called mitophagy, has emerged as a crucial determinant at the interface between tumor cells and TME [15]. Growing evidence shows that mitophagy pathways are key regulators of mitochondrial mass, mitochondrial dynamics, redox homeostasis, metabolic reprogramming and cancer cell survival or death. Mitophagy would represent a metabolic adaptation mechanism capable of fostering the survival of cancer cells within an otherwise unfavorable microenvironment. According to this, it has been observed that many proteins involved in mitophagy are dysregulated in some tumors [16]. In addition to cancer cells, the mitophagy of stromal cells would promote tumor progression by providing essential compounds in the metabolism of cancer cells [17]. In accordance with the above, the induction of mitophagy by anticancer drugs could contribute to resistance to therapy, a hypothesis supported by the fact that the inhibition of mitophagy was able, in some models, to re-sensitize cancer cells to pharmacological treatment [18]. On the other hand, a reduced mitophagy increases mitochondrial reactive oxygen species (ROS) levels. The consequent oxidative stress, through HIF-1α stabilization, can promote the Warburg effect, favoring tumor progression even in normoxia conditions. The Warburg effect, referred as to the production of ATP through glycolysis rather than through OXPHOS, can in turn result in lactate production and contribute to the acidification of TME, indirectly regulating tumor invasion and metastasis [19] as well as drug resistance [20]. Hence, strategies aimed at buffering the TME pH have also been proposed in both preclinical [21] and in clinical studies (NCT01198821) [22] whereas strategies aimed at the control of mitophagic process are still lacking.

An additional point to be considered concerns the epigenetic regulation of autophagy in cancer. Several miRs have been shown to modulate the autophagy mechanisms and the levels of some miRs regulating autophagy have been found altered in the oncology context [23]. Importantly, dysregulation of these miRs has been observed both in tumor and stromal cells, and the cross talk between tumor and stromal miRs can determine the characteristics of the TME. Of note, the miRs present in the TME are either free or enclosed within vesicles and/or exosomes, and can act both as tumor suppressors and as activators [24].

### 1.3. Sex Differences in Cancer

The third actor considered herein is related to the sex/gender disparity observed in many forms of cancers [25,26]. In fact, significant sex/gender differences in cancer incidence, progression, and response to therapy have been reported [26,27,28,29,30]. Importantly, differences in the clinical outcome have recently been reported for many types of tumors [30,31,32]. On the other hand, several observations were made with regard to the role of the sex of the patient on the activation of the autophagy pathway [33]. These differences are partly related to the effects of steroid hormones, especially estrogen. Moreover, some studies reported that, irrespective of steroid hormones, sex chromosomes could play a role per se. Indeed, XX cells appear more prone to autophagy than XY ones: cells from females could survive better than cells from males in unfavorable environmental conditions thanks to a higher autophagic propensity [34,35,36,37,38]. A detailed review has recently been published addressing sex differences in a range of autophagy-mediated diseases, including cancer. The authors correctly state that a clinical approach that takes into account differences related to the sex of subject (and gender, the socio-economical, nutritional aspects characterizing a subject) is a first step towards precision medicine [39]. Cancer therapy may be the branch of medicine closest to this innovative clinical approach.

Epigenetics is also part of this scenario. In fact, a sex-specific regulation of miRs has been suggested in many normal and pathological tissues [40]. A direct action of sex hormones, especially estrogen and progesterone, as well as of sex chromosomes [41], has been hypothesized. The X chromosome contains numerous miRs (about 10% of the total) [42] whereas the Y chromosome seems to contain four miRs only. The incomplete inactivation of one of the two X chromosomes in female cells has been proposed to represent one of the possible mechanisms underlying the sexual dimorphism observed in the expression of some X-linked miRs [43,44,45].

In summary, not only are the cancer cells themselves very heterogeneous, but the TME and the sex of the hosts contribute greatly to increasing the complexity of the tumor. On this basis, it has been proposed that the patient’s sex and gender should be considered as an additional variable in the field of oncology, also in reference to antineoplastic treatment [26,46].

In this review, we present the “state of the art” of current knowledge on the role played by autophagy in the crosstalk between the tumor cell and the microenvironment, taking into account sex differences. The roles played by steroid hormones, in particular estrogen, and miRs are considered, mainly focusing our attention on four paradigmatic not-related forms of cancer: colon cancer, melanoma, lymphoma, and lung cancer.

## 2. Estrogen and Estrogen-Mediated Autophagy as Tumor and TME Key Determinants

### 2.1. Estrogen and Estrogen Receptors

The biological effects of estrogen, the main female sex hormone, are mediated by the binding of estrogen to estrogen receptors (ERs) [47], which, besides being a crucial determinant in reproductive processes, also plays a role in many physiological processes in a number of tissues, organs and systems, such as the central nervous, cardiovascular and immune systems. In contrast to women, men are largely dependent on the local synthesis of estrogens in extragonadal target tissues, i.e., via the cytochrome P450 enzyme aromatase that catalyzes the conversion of androgens to estrogens.

ERs belong to the steroid hormone superfamily of receptors mostly localized in the cell nucleus. In addition, ERs have also been detected at the cell surface, in the cytoplasm and at the mitochondrial level. Two different types of ERs have been identified so far: the estrogen receptor alpha (ERα) [48,49], and the estrogen receptor beta (ERβ) [50,51].

The biological effects of estrogens are primarily carried out by the modulation of gene transcription. In fact, after binding to estrogen, ERs dimerize and migrate into the nucleus, where they bind an Estrogen Response Element (ERE). However, further estrogen signaling pathways have been demonstrated. Actually, as reported above, ERs can also be detected at the plasma membrane (mER), where they are embedded in lipid rafts (sphingolipid enriched membrane microdomains) [52]. These ERs can ignite non-genomic pathways such as the prompt activation of the mitogen-activated protein (MAP) kinase signaling pathway [53,54]. Another mER, structurally unrelated to the other ERs, but capable of inducing rapid non-genomic signals, has also been identified and named G protein-coupled receptor 30 (GPR30) [55].

### 2.2. Estrogen and Autophagy

Estrogen plays a critical role in mediating many of the physiological cell functions, such as growth, differentiation, metabolism, and death. In initial studies in animal models, an increase in lysosomes and in degrading organelles was observed to be induced by castration, suggesting that sex hormones could affect autophagy [33,56]. These data have more recently been confirmed, demonstrating that the estrogen signaling network can stimulate autophagy [57,58] or, conversely, decrease overstimulated autophagy [59,60,61].

**Figure 2:**
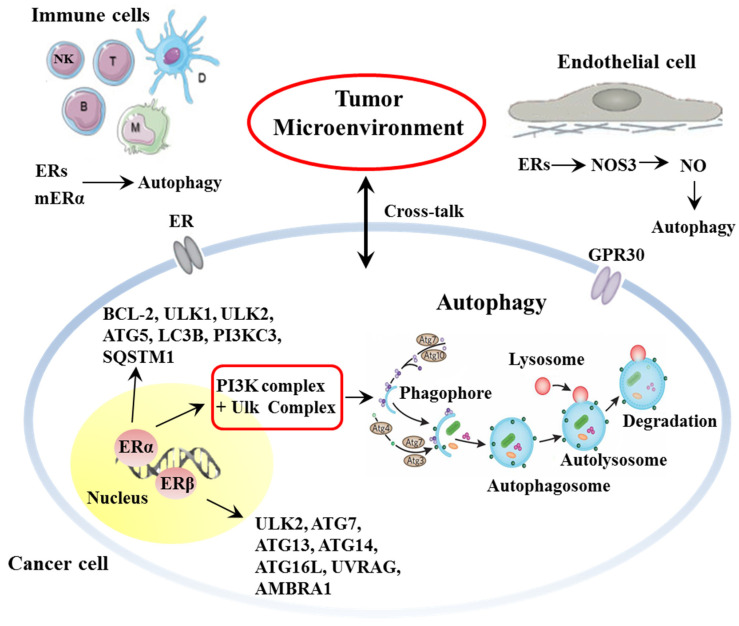
Simplified scheme representing the role of ER in autophagy modulation in cancer and TME.

ERα and ERβ seem to play a role in regulating autophagic processes, both in cancer and TME, via a delicate and tricky process aimed at controlling cell homeostasis (Figure 2).

At the genomic level, ERα has been shown to regulate the transcription of B-cell lymphoma 2 (Bcl-2) and Unc-51-like autophagy activating kinase (ULK)-1, two key molecules that determine cell fate [39,62,63]. ERα thus seems able to regulate phagophore induction by controlling many autophagy genes in the ULK complex and the phosphatidylinositol 3-kinase (PtdIns3K) complex 1 at the transcriptional level. Bioinformatics analysis confirmed these data, suggesting that both ERα and Erβ, at the transcription level, can regulate several autophagy genes [64]. Twelve autophagy genes were observed as regulated by ERβ, including ULK2, ATG7, ATG13, ATG14, ATG16L1, UVRAG, and AMBRA1, and 19 autophagy genes appeared as regulated by ERα, such as ULK2, ATG5, LC3B, PIK3C3, and SQSTM1. Moreover, Felzen and co-workers [65] observed that ERα was able to mediate the BAG3-non-canonical autophagy pathway. In another study, Yang and co-workers suggested that ERβ was involved in the induction of autophagy by the inhibition of the PI3K/AKT/mTOR pathway and the activation the AMPK pathway [66].

Several further aspects are of great relevance in this context and still need to be investigated in more detail. For instance, the main issues are: (i) the role of mitochondrial ERs that have been detected in many human cell types [67,68,69]; (ii) the role of estrogen in selective mitochondrial autophagy (mitophagy); (iii) the role of ERs localized in lysosomes [70,71] that could be involved in autophagy induction upon fusion with autophagosomes. Hence, the activity of estrogen and its receptors in regulating cell fate, i.e., survival by autophagy or death by apoptosis, appears to be pivotal.

A final general point to be considered here is represented by the fact that estrogen has been widely demonstrated to play an essential part in the function of TME and autophagy modulation [72]. In endothelial cells, ERs, after binding with their ligands, activate nitric oxide synthase 3 (NOS3) [73], leading to the synthesis of nitric oxide (NO), which can inhibit the expression of mTOR and induce autophagy [74].

As for immune cells, they are known to actively participate in TME formation, maintenance and activity. Autophagy modulates the integrity and function of several immune cell types, such as natural killer (NK) cells, macrophages, dendritic cells, and T- and B-lymphocytes [75]. ERs are expressed across multiple immune cell population and modulate their activity [76,77]. Lymphocytes express ERs even at their surface [78], allowing a prompt response to their agonists and, thus, taking part in the modulatory role of TME. In particular, estrogens are involved in regulating neutrophil chemotaxis and proliferation [79] and DCs differentiation [80]. Estrogens, in particular 17-β estradiol (E2), have different effects depending on their concentration. At high levels (e.g., pre-ovulatory phase or pregnancy), E2 mainly exerts anti-inflammatory effects, inhibiting the production of pro-inflammatory cytokines, such as TNF-α, IL-1β and IL-6, and inducing the expression of anti-inflammatory cytokines, such as IL-4, IL-10 and TGF-β, also favoring Treg function [81]. At lower levels, E2 induces TNF-α, IFN-γ and IL-1β production and NK cell activity. Moreover, E2, both at high and low concentrations, induces antibody production by B cells. It is worth noting that ERα and ERβ play opposite roles in immune function, with pro- and anti-inflammatory activity, respectively. Hence, the effects of E2 depend not only on its concentration, but also on the ER subtype expressed by target cells [82,83].

Together, these studies underline the crucial role of the ER/E2 axis in the tumor immune microenvironment [84]. However, the possible immunotherapeutic implications of estrogen targeting in cancer patients is still a matter of study and debate. For example, it has been observed that immunotherapy with immune checkpoint inhibitors (ICI), able to improve the immune response against cancer, is more effective in men than in women. It can be hypothesized that the immune response, constitutively higher in women than in men, cannot be bolstered further [29,85].

### 2.3. Estrogen and Cancer

The activity of estrogen in regulating autophagy appears to be pivotal in the onset and progression of many types of tumors. Below, we will briefly describe four paradigmatic examples of cancers such as colon cancer (CC), lymphoma, melanoma and lung carcinoma (LC), and their strict interplay with estrogen (Table 1).

#### 2.3.1. Colon Cancer

Epidemiological studies reported that men display a higher prevalence of colon cancer. However, ascending colon cancer is more frequent in women, and women aged 18–44 with colon cancer had a better prognosis compared with men of the same age and with post-menopausal women [91]. Upregulated expression of ERβ1 (the only fully functional isoform of ERβ) in colon cancer is associated with an improved survival outcome [92] while the ERβ expression decreases progressively with the worsening of the stage and grade of colon cancer [93]. The mechanisms underlying this activity are still to be defined in detail. In a recent study, Wei and co-workers [86] demonstrated that ERβ suppressed proliferation of a colon cancer cell line, i.e., HCT16 cells, without any effect on cancer cell apoptosis. In particular, ERβ ligation led to G1-S phase arrest in HCT116. The authors demonstrated that ERβ induced CylinD1 degradation via mTOR-dependent autophagy. Additionally, ERβ induced autophagic cell death via BNIP3, a member of the Bcl-2 protein family that can modulate the permeability state of the outer mitochondrial membrane. A further aspect to be taken into consideration in colon cancer is the treatment with 4-hydroxytamoxifen, a selective ER modulator widely used in the therapeutic and chemo-preventive treatment of breast cancer, that caused the degradation of KRAS, a protein whose gene appeared often mutated in colon cancer, through autophagy preventing cancer cell growth [87].

The role of ERα in colon cancer cell fate is still poorly studied. It has been observed, however, that this receptor is poorly expressed in colon cancer cells [94].

#### 2.3.2. Melanoma

Although melanoma is classically considered a non-hormone-dependent tumor, a growing line of evidence supports a direct correlation between estrogen and melanoma growth and progression [95,96]. Interestingly, the skin is able to produce estrogens [95] since the aromatase, i.e., the enzyme that convers androgens to estrogens, is expressed in melanoma tissues [97]. Melanoma incidence is higher in men than in women but, conversely, women show a better outcome in comparison to men [98]. ERα is the main ER in human skin; however, ERβ is the predominant ER in melanocytic lesions, and its expression decreases in melanoma progression, supporting its role as a tumor suppressor [99,100,101,102]. It is worth noting that the expression levels of ERβ have been found to be lower in men than in women in both melanoma and healthy tissues, which is in line with sex differences in melanoma patient survival [103,104]. Although estradiol (E2, the most important human estrogen) has been observed to be able to hamper melanoma cell proliferation, clinical trials failed to demonstrate survival advantages [105]. At variance, in vitro studies with ERβ agonists have suggested that they could inhibit the proliferation of melanoma cells harboring the NRAS mutation, an important gene of the RAS family [88], indicating that ERβ might impair melanoma onset through inhibition of the PI3K/Akt pathway. Interestingly, Tamoxifen, a selective estrogen receptor modulator, in combination with the antioxidant curcumin, has been demonstrated to induce autophagy and apoptosis in melanoma cells [89]. However, to date, the role of autophagy in this process should be investigated in more detail.

#### 2.3.3. Lymphoma

In general, adult men are slightly more likely to develop lymphoma and have a poorer prognosis and survival than women [30]. Even though the underlying reasons have been scarcely understood, estrogens seem to play a crucial role in these sex differences [90]. Accordingly, the female advantage begins with puberty, increases until menopause, and then declines [30]. In particular, ERβ activation by selective agonists was found to inhibit the growth of non-Hodgkin lymphoma (NHL, the significantly more frequent form of lymphoma, constituting about 90% of cases), mainly by reducing cell proliferation [106,107]. Furthermore, ERβ agonists were also able to prevent lymphoma vascularization and dissemination in mice [106]. In addition, at least in vitro, ERβ-induced damage regulated autophagy modulator 2 (DRAM2)-mediated autophagy has been associated with a reduction in cancer cell proliferation in Hodgkin lymphoma (HL) cells [78].

#### 2.3.4. Lung Carcinoma

In the past three decades, lung carcinoma incidence and mortality were significantly decreased in men, whereas they increased among women. In particular, premenopausal women are more frequently diagnosed with advanced stage tumor, have less differentiated tumors, a higher number of distant metastases and worse prognosis than both men and postmenopausal women [108,109,110,111]. These different manifestations of LC appear to be at least partially due to estrogen effects. Several points have in fact emerged from the literature. For instance, non-small cell lung cancer (NSCLC) has now been recognized as an ER-positive cancer. Both ERα and ERβ have been identified in NSCLC [112,113]. ERβ is the predominant type of ER in NSCLC, and ERβ overexpression correlates with poor prognosis [114,115]. Notably, E2 has been observed to induce NSCLC cell proliferation and tumor growth, mainly from activation of cAMP, MAPK and AKT signaling by non-genomic pathways. Furthermore, E2, which together with high aromatase expression has been reported to be more elevated in tumor compared to healthy lung [112,116,117], induces the expression of c-Myc, Cyclin D, and the inhibitor of differentiation (Id) proteins by genomic pathway, leading to cell cycle progression [118,119]. Moreover, mitochondrial ERβ has been reported to play anti-apoptotic effects in NSCLC cells [120].

A link between ERβ and autophagy in LC has been suggested by Qiu and co-workers in a recent study [121]. They observed that fragile-site associated tumor suppressor (FATS), a novel oncogene involved in LC, was significantly downregulated in NSCLC tissues compared with adjacent normal tissues and was associated with the survival of NSCLC patients. The authors demonstrated that the presence of the tumor suppressor FATS in NSCLC cells led to apoptosis by inducing pro-death autophagy. FATS was shown to function as a suppressor of polyamine biosynthesis by inhibiting ornithine decarboxylase (ODC) at the protein and mRNA levels, and this mechanism was dependent on ERβ. In fact, FATS binds to ERβ and translocates to the cytosol, leading to ODC degradation [121].

The role of ER in autophagy induction in NSCLC has further been supported in a study by Zhang and coworkers [122] that analyzed the effect of genistein, a phytoestrogen that acts as an estrogen receptor agonist, in NSCLC cells. Treatment with genistein significantly enhanced radio-sensitivity of NSCLC cells by increasing apoptosis, which occurs as a result of the inhibition of cytoplasmic Bcl-xL distribution, and autophagy, by hindering the interaction of Bcl-xL and Beclin-1.

## 3. Sex-Specific Epigenetic Control: MicroRNAs and Autophagy

MicroRNAs are potent epigenetic regulators of gene expression, capable of post-transcriptional gene control. They, acting on the stability or translation of mRNA, modulate a number of targeted proteins in the cell. Experimental evidence has clearly demonstrated their impressive role in cancer development and progression. More than 2000 miRs were discovered, with tumor suppressor or oncogenic roles, thus contributing to various stages of cancer formation and progression, and eventually favoring resistance to treatment [123]. A great number of works have described miR biogenesis and transcriptional regulation and we refer to them for exhaustive explanation [124,125]. miRs were shown to modulate levels of several key proteins of the autophagy pathway, from the upstream signaling of vesicle nucleation to later stages of autolysosome degradation. Autocrine and paracrine signals, respectively representing pro- and anti-oncogenic mechanisms, can be sustained by miRs, either bound with some specific proteins or loaded and released by extracellular vesicles, favoring communication among tumor cells, and between tumor and stroma cells as well as tumors and distant compartments [126]. Sometimes, as stated above, this cross talk is complicated by hormonal regulation, mainly based on estrogen, that might influence autophagic or apoptotic responses. Although miR action favors cancer cell adaptation to host responses against tumor, including immune and pharmacological responses, some miRs are able to modulate the autophagic pathway to achieve the desired final effect of apoptotic program activation, finally reducing the tumor burden. This effect is essential for silenced tumor suppressor miRs that, when re-expressed, can act in association with anticancer drugs, favoring therapeutic response (Table 2). Finally, as noted above, the X chromosome contains a high number of miRs (about 10% of the total) and the incomplete inactivation of one of the two X chromosomes in female cells could lead to the sexual dimorphism observed in the expression of some X-linked miRs [43,44,45]. Only four miRs have been so far identified on the Y chromosome [42].

### 3.1. Colon Cancer

Colon cancer can, in part, be recognized as a complication of long-term colon inflammatory diseases, such as Crohn’s disease (CD) and ulcerative colitis (UC), primarily due to an abnormal immune response in which dysfunction of autophagy has been considered an important contributing factor [139]. In this microenvironment, some miRs are inhibitors of autophagy by regulating different ATG proteins, eventually influencing the innate immune response. Sex hormones might participate in miR-specific regulation, in turn contributing to the regulation of autophagy responses. For instance, miR-142-3p affects the immune microenvironment in CD by direct targeting of IL-8 mRNA expression and inhibition of inflammatory bowel disease protein 1 (IBD1)-dependent autophagy [140]. However, in the tumor context, the same miR was down modulated by ERβ expression in colon cancer cell lines, through an estrogenic binding site upstream of the miR-142-3p locus [141]. Evolution of CD in CC and its progression are characterized by increased hypoxic microenvironment advantaging glycolysis in parallel with increased autophagy activation to avoid tumor cell death. In this condition, the efficacy of therapeutic actions is particularly complicated, possibly favoring tumor cell metastatization.

The autophagic flux in human colon cancer cell lines grown in hypoxia is regulated by HIF-1α expression. HIF-α, in turn, positively regulates miR-210, which, by limiting the expression of Bcl-2, induces cell autophagy, eventually contributing to radio resistance [127].

Furthermore, some miRs control, at different levels, the autophagy activation, favoring drug sensitivity of CC cells. MiR-22, which inhibits estrogen signaling by direct targeting of ERα mRNA, can increase cell sensitivity to 5-fluorouracil (5FU) inhibiting autophagy by regulation of the B-cell translocation gene 1 (BTG1), a regulator of cell growth and differentiation [128,142].

It is now clear that chemotherapy efficacy is also associated with a good immune response, whereas autophagy determines immune evasion. Indeed, both apoptotic- and autophagy-dependent cell deaths are potent activators of the adaptive immune response through the release of the Damage-Associated Molecular Patterns (DAMPs). DAMPs are recognized by receptors of dendritic cells and finally favor the development of productive microenvironments with expression of cytokines that are capable of promoting immune response and, more importantly, of activating the immune cell mediated apoptotic program [143]. The opposite action of miR-27a, induced by drug treatments that inhibits DAMPs’ release eventually supporting autophagy activation and resistance, should also be considered. In a favorable microenvironment, the low expression of miR-27a, which indirectly regulates ERα expression and hormone responsiveness [144], is associated with a good prognosis in CC, which responds with immunogenic cell death to chemotherapy [129].

A final point to be considered is that CC TME is characterized by a massive presence of cancer-associated fibroblasts (CAFs) that, interacting with tumor cells, secrete extracellular matrix components and growth factors that regulate immune response. It was evidenced that CAFs were capable of negatively modulating the expression of diverse autophagy-related proteins in tumor cells through miR-31. Importantly, this interaction positively affected the sensitivity of CC cells to radiation therapy [13].

### 3.2. Melanoma

Autophagy activation is important in the protection of normal epidermis from ultraviolet irradiation. In these stress conditions, autophagy is activated to avoid the detrimental effect of photo-aging, a phenomenon that is associated with diverse pathologies, including skin cancer. Abnormality in autophagy was related to the expression of miR-23a, which caused premature senescence of epidermal fibroblasts. This event was avoided by antagomiR-mediated inactivation of miR-23a, with the consequent stimulation of ultraviolet-dependent autophagy protecting human fibroblasts from premature senescence. The limiting factor in the rescue of fibroblast senescence was the AMBRA1 protein, identified as an miR-23a target [145]. The same miR works as tumor suppressor in melanoma, being down-modulated in serum and tissues of patients with metastatic melanoma, which is, in turn, associated with metastasis formation. When re-expressed in A2058 and A375 melanoma cell lines, invasion capability was strongly reduced by the miR-23a-dependent ATG12 targeting and relative autophagy inhibition [130].

One prerogative of the tumor setting is the high grade of adaptability of tumor cells to extreme microenvironments, eventually becoming capable of inducing the autophagy process through miR regulation [146,147,148,149]. An example of this is miR-26a, whose levels drop in melanoma cells treated with dabrafenib (a drug targeting a mutated version of the gene BRAF (V600E)). Beyond pharmacological treatment, the effect on autophagy activation by miR-26a silencing was a consequence of the deficiency in the endothelial recovery, associated with the absence of miR-26a dependent differentiation of mesenchymal stem cells in functional endothelial cells. This deficiency, reducing tumor blood flow, favored the Warburg effect in the TME [150]. Indeed, miR-26a re-expression sensitized melanoma cell lines to dabrafenib, targeting the high mobility group box 1 (HMGB1)-dependent autophagy, and finally, engaging the apoptosis program [131].

Different miRs induce autophagy by silencing the expression of proteins associated with the mTOR signaling pathway. Licochalcone A is a phytoestrogen of the flavonoid family (LCA), with an antioxidant protectant role, that has been shown to exert anticancer activity by inducing autophagy and apoptosis. In A375 and B16 melanoma cell lines, this effect was associated with up-modulation of miR-142-3p and decreased expression of its target, the Ras homolog enriched in brain (Rheb) protein, with consequent suppression of mTOR signaling activation [132]. These data seem to suggest a reappraisal of phytoestrogens in the clinical control of melanoma.

More generally, the recent elegant review by Dika and colleagues [151] describes in some detail the influence of ERs on melanoma, also evaluating sex disparity for this form of cancer, whose incidence continues to increase globally. In particular, these authors claim that there is a direct correlation between estrogens and the female survival advantage, which seems to be abrogated in the postmenopausal period, during which estrogen levels decrease. Either a network involving ERs and miRs, characterized by a direct effect of estrogen on miR expression or, at variance, revealing a direct regulatory effect of miRs on ER expression, seems to take place. For example, estrogen signaling triggered by ERα appears to inhibit the levels of miR-21, miR-26a, miR-140, miR-181b and miR-206, whereas it upregulates miR-190a, miR-191, miR-203 and miR-425. In addition, ERβ signaling participates in these pathways by downregulating miR-17, miR-30a, miR-200a and miR-200b, and inducing the overexpression of miR-23b, miR-24-1 and miR-27b. These data appear to underscore the complex anatomy of estrogen/TME crosstalk as a determinant of the melanocyte/melanoma control pathway.

### 3.3. Lymphoma

Currently, miR involvement in the regulation of autophagy in lymphoma has barely been investigated. Some miRs have been shown to have a tumor suppressor role and their expression was commonly silenced by epigenetic regulation, indicating a pathogenetic mechanism for lymphoma development. In B-cell lymphoma, miR-342 is associated with ERα expression. It is localized on chromosome 14 in the third intron of its host gene Enah/Vasp-like (EVL), a regulator of actin cytoskeleton remodeling, which is essential for cell migration ability. In primary samples of B cell lymphoma, miR-342 was found to be silenced by hyper methylation of the promoter region of the EVL gene, and 5-AzadC treatment resulted in re-expression of miR-342-3p and EVL protein. Furthermore, transfection of synthetic miR-342-3p reduced cell proliferation and inhibited autophagy in SU-DHL-16 cells, eventually increasing cell death. The microtubule-associated proteins 1A/1B light chain 3B (MAP1LC3B), a precursor of the autophagy functional marker light chain 3 (LC3)-II, and DNA methyltransferase-1, were validated as targets of miR-342-3p. The negative regulation of the DNA methyltransferase-1 by miR-342 induced DNA hypo methylation and reduced lymphoma malignancy [133,152,153].

T cell lymphoma is a non-Hodgkin’s lymphoma originating from T cells characterized by high heterogeneity and malignancy. The absence of an effective cure for this pathology indicates that novel strategies of treatment must be developed. Therefore, it has been proposed that targeting autophagy might reconstitute sensitivity to doxorubicin in this disease. Indeed, in this lymphoma, miR-449a is frequently silenced. This miR acts as a tumor suppressor in human glioblastoma and hepatocellular carcinoma, and it suppresses anti-estrogenic treatment resistance in human breast cancer cells [154]. Expression of miR-449a attenuated autophagy and induced apoptosis in T cell lymphoma cells. Direct targeting of ATG4B mRNA induced this effect. In vivo experiments also demonstrated growth inhibition of T cell lymphoma xenograft tumors in nude mice, which was associated with activation of the pro-apoptotic program, as indicated by increased cleaved Caspase-3 and PARP [134].

### 3.4. Lung Carcinoma

Among other factors, the role of miRs and their altered and deregulated levels appear to be critical in lung cancer. If intercepted in time, by specific serum assays, they could represent a fundamental tool for increased diagnostic/therapeutic opportunities, especially in drug resistant microenvironments that are responsible for the untreatable metastasizing events. NSCLCs that are resistant to gefitinib, a selective EGFR tyrosine kinase inhibitor, are characterized by low expression of miR-153-3p, which is inversely correlated with the autophagy marker LC3B. ATG5 is a direct target of miR-153-3p, and in gefitinib-resistant NSCLC, the ATG5 level was high, in parallel with autophagy activity. Accordingly, because of miR-153-3p restored expression, in lung cancer cell lines, autophagy can be reduced and apoptotic cell death strengthened [135]. Of note, evidence has been reported of a functional interaction between the ER and the EGFR pathways in NSCLC. In particular, combined targeting of the estrogen receptor and the epidermal growth factor receptor in NSCLC has demonstrated the enhancement of their antiproliferative effects [155]. This could open new scenarios in the understanding of either possible alternative pathogenetic mechanisms or therapeutic options in men and women. In fact, some miRs can modify the therapeutic performance of specific drugs, removing the protective autophagy in lung cancer cell lines and inducing apoptotic programs. In this context, the X-linked miR-106a was indicated in ULK1 targeting, being essentially down-modulated after treatments with tyrosine kinase inhibitors and other chemotherapeutic agents. MiR-106a silencing, beyond blocking the apoptotic program, increased the level of ULK1, favoring a protective autophagy [136]. In addition, it was proposed that the ERα transcriptional programs could involve miR-106a activity and function [156].

Cisplatin resistance is the major problem in the treatment of advanced lung cancer. The naturally occurring triterpenoid quinone, a compound with antiestrogenic activity named pristimerin [157], induced enhanced chemosensitivity to cisplatin treatment. The mechanism underlying this increased sensitivity was investigated in A549 and NCIH446 lung cancer cell lines and in in vivo experiments. The authors found that pristimerin, possibly thanks to its antiestrogenic activity, was capable of synergizing with cisplatin to inhibit the miR-23a/Akt/glycogen synthase kinase 3β signaling pathway, thus suppressing autophagy. These inhibitory events were associated with increased tumor cell death by apoptosis [137].

Sex dimorphism in the cell response to drugs has been suggested to be, at least in part, due to female sex-biased expression of miRs, e.g., the X chromosome-encoded miRs potentially escaping X chromosome inactivation (XCI). A few X-linked miRs have been analyzed in some detail but, unfortunately, their activity is still far from being well elucidated. Among them, the X-linked miR-384 was found to be down-regulated in lung cancer. Indeed, when expressed in lung cancer cell lines and implanted in nude rats, miR-384 favored autophagy and apoptosis by targeting the TME Collagen α-1(X) chain (COL10A1), while it inhibited proliferation [138]. This suggests a further role of the TME/miR/cancer interplay.

Hence, as a rule, a great deal of work still appears to be necessary in order to elucidate the possible redundant expression of X-linked miRs, mainly those with tumor suppressor roles that are commonly silenced by epigenetic regulation. These miRs, if maintained in an activated state, might partially explain the differences in the pathogenetic mechanisms of cancer development between the two sexes.

## 4. TME–Cancer Cells Crosstalk as Possible Target for Cancer Growth Control

As described above, autophagy is an essential process contributing to cell cytoprotection and homeostasis. It is strictly related to the metabolic supply by intracellular milieu as well as by extracellular constituents, e.g., those composing the TME. Several results obtained both in vitro and in in vivo mouse models of cancers showed that autophagy could impair the growth of benign lesions, but could favor that of malignant ones [158]. Indeed, tumor initiation is impaired by the autophagic actions, and Beclin1 was reported as a tumor suppressor in breast, ovarian, and prostate cancers. In addition, autophagy can reduce tumor metastases by down-regulating some transcription factors involved in the EMT transition, such as Snail and Slug, in turn favoring E-cadherin cell adhesion molecule expression [159]. Although several data suggested autophagy as a possible therapeutic target, the double action exerted by this process in cancer made it difficult to select key factors and biological processes to aim at, and the quantity of apparently controversial data indicates the relevance of considering the context-associated roles of autophagy [160]. Generally, in cancer studies, the induction of autophagy could represent a useful action leading to the blocking of cell cycle progression and, in turn, of cell proliferation. However, as reported above, this could lead to the so-called dormant cancer cells giving rise to relapsing disease.

In the last decade, the identification of novel therapeutic drugs led to significant results for several tumor types, thus improving patients’ survival. Nonetheless, drug resistance still represents a major challenge, which reinforces the need to further dissect the molecular abnormalities underlying cancer progression. Indeed, a better comprehension of the process is required to solve the debate around inhibiting or inducing autophagy. Actually, the complexity of this functional pathway was shown by many different studies. In vivo studies in mouse models supported the possible therapeutic value of inducing autophagy, as the loss of one Beclin1 allele favored the development of tumors. However, this result was not confirmed in cancer patients, where BECN1 did not seem to represent a key player [161], and autophagy induced by antitumorigenic drugs demonstrated protective results for tumor cells [162].

According to some negative results associated with induced autophagy, the majority of the studies are now focused on its inhibition. At present, many different drugs have been developed for experimental research, but they still lack confirmed specificity and suitability in vivo. The main aim of these studies was precisely the idea of hindering the “feeding” of cancer cells via TME, an important source of metabolites and nutrients [163].

Looking at the US National Library of Medicine (search dated May 2021), located at ClinicalTrials.gov, using the search terms Cancer and Autophagy, we found 84 studies, most of them based on hydroxychloroquine alone or in combination. Eleven studies are terminated, with their results available, but the number of recruited subjects was generally low and insufficient data were collected. In addition, due to lack of therapeutic improvement with respect to controls, several studies were interrupted ahead of schedule. However, it cannot be ruled out that the antitumor effects shown by chloroquine and its derivatives are not directly linked to the induction of autophagy on cancer cells. In fact, very interestingly, some in vitro and in vivo studies demonstrated that the tumor cell sensitivity to quinacrine [164] or hydroxychloroquine [165], two chloroquine derivatives, were strongly dependent on TME.

Among the autophagy-related proteins, we should also consider mTOR, the mechanistic target of rapamycin that has been shown to represent a key target, specifically its inhibition associated with autophagy induction. Rapamycin and its derivatives, such as everolimus and sirolimus, have been demonstrated to inhibit proliferation in several different human tumors [166]. Recent evidence also showed the higher effectiveness of combination treatment of mTOR inhibitors with hydroxychloroquine [167]. Although everolimus appears capable of affecting the integrity and function of some lymphocyte subsets, its effects on immune cell components of TME are still a matter of debate [168]. In fact, with the growing relevance of immunotherapy in the fight against cancer, the interplay between autophagy and immune responses, either in general or associated with TME, appears to be of great interest. The induction of both innate and adaptive immune responses might reinforce or reduce the therapeutic effects of immunotherapies [75]. Whenever completely clarified, all these connections might provide some novel therapeutic targets that will possibly be translatable into effective therapeutic strategies [169].

## 5. Conclusions

As both the microenvironment and sex, e.g., hormones and sex chromosomes, as well as autophagy inhibition or bolstering appear to affect the responsiveness of tumor cells to therapy, the development of novel experimental models appears to be an indispensable tool to develop increasingly targeted and personalized therapies. These models should recreate the physiological and mechanical cues typical of cancer microenvironment, recapitulating the relationship between tumor cells and TME and possibly reproducing sexual dimorphism [39]. Recent advances in three-dimensional (3D) modeling, also using chips and microfluidic devices [170,171], provide us with the opportunity of studying the biology of TME. Understanding the cellular and molecular mechanisms that govern the complex interactions between tumor cells and microenvironments could contribute to the progress of therapeutic strategies that encompass the biological complexity of a tumor by reducing the gap between translational research and clinical practice.

Since autophagy has variable effects on tumor cells, depending on the context and stage, the role of TME in providing further survival options to cancer cells could be pivotal. However, the relevance of the microenvironment is due to its heterogeneous composition where signals, e.g., hormones, immune-inflammatory cells, recyclable materials, and ECM proteins, are paired with the origin of cancer cells not only by a histotypical point of view or by tumor staging, but also by a novel and fascinating point of view: the “sex” of the cell. Based on the previously published observations on the disparity of cancers between women and men in terms of incidence, progression and response to therapy [19], this field could represent a novel challenge and a first key step for the development of precision medicine.

Systematic research on sex differences in cancer biology is needed to evaluate sex-specific dosages of anticancer drugs, especially for those that show significant pharmacokinetic differences in the two sexes, thereby improving the efficacy/toxicity ratio. In addition, for tumors or tumor subtypes with significant gender differences in incidence and outcome, sex-specific therapeutic approaches should already be considered. A better understanding of the molecular-pathogenetic mechanisms underlying these differences could guarantee effective improvement in a personalized and tailored approach that is of great relevance in the fight against cancer.

## Figures and Tables

**Table 1:** Examples of Estrogen Receptor β mediated effects on autophagy modulation.

Cancer	Effects of ERs on Autophagy	References
Colon cancer	ERβ induced autophagy through: mTOR inhibition BNIP3 degradation of KRAS	[86,87]
Melanoma	ERβ induced autophagy through: inhibition of the PI3K/Akt pathway	[88]
Hodgkin Lymphoma	ERβ induced autophagy through: DRAM2 expression	[78]
Lung carcinoma	ERβ induced autophagy in NSCLC cells through: binding to FATS hindering the interaction of Bcl-xL and Beclin-1 (effect mediated by genistein, a selective agonist of ERβ)	[89,90]

ERβ, estrogen receptor beta; mTOR, mammalian target of rapamycin; BNIP3, Bcl-2 and adenovirus E1B 19-kDa-interacting protein 3; KRAS, Kirsten Rat Sarcoma; PI3K/AKT, Phosphatidylinositol-3-Kinase/Protein Kinase B; DRAM2, damage regulated autophagy modulator 2; NSCLC, non-small cell lung cancer; FATS, fragile-site associated tumor suppressor; Bcl-xL, B-cell lymphoma-extra Large.

**Table 2:** MicroRNAs involved in autophagy/apoptosis regulation.

Cancer	microRNAs	Targeted Proteins	Effect on Tumor Cells	References
Colon Cancer	miR-210	Bcl-2	Induces autophagy and radioresistance	[127]
	miR-22	BTG1	Inhibits autophagy and promotes apoptosis	[128]
	miR-27a	Calreticulin	Inhibits autophagy and apoptosis	[129]
Melanoma	miR-23a	ATG12	Inhibits autophagy and reduces invasiveness	[130]
	miR-26a	HMGB1	Inhibits autophagy and induces apoptosis	[131]
	miR-142-3p	Rheb	Induces autophagy and apoptosis	[132]
Hodgkin Lymphoma	miR-342-3p	MAP1LC3B, DNMT1	Inhibits autophagy and induces cell death	[133]
	miR-449a	ATG4B	Inhibits autophagy and induces apoptosis	[134]
Lung Carcinoma	miR-153-3p	ATG5	Inhibits autophagy and induces apoptosis	[135]
	miR-106a	ULK1	Inhibits autophagy and induces apoptosis	[136]
	miR-23a	PTEN	Induces autophagy and inhibits apoptosis	[137]
	miR-384	COL10A1	Induces autophagy and apoptosis	[138]

Bcl-2, B-cell lymphoma 2; BTG1, B-cell Translocation Gene 1; ATG12, Autophagy-related 12; HMGB1, High mobility group box 1; Rheb, Ras homolog enriched in brain; MAP1LC3B, Microtubule-associated protein 1A/1B light chain 3B; DNMT1, DNA Methyltransferase 1; ATG4B, Autophagy-associated 4B; ATG5, Autophagy-related 5; ULK1, Unc-51-like autophagy activating kinase 1; PTEN, Phosphatase and tensin homolog; COL10A1, Collagen α-1(X) chain.

## Data Availability

The data presented in this study is available within the article.

## Abbreviations

5FU	5-fluorouracil
AKT	protein Kinase B
AMPK	adenosine monophosphate-activated protein kinase
ATG	autophagy gene
Bcl-2	B-cell lymphoma 2
Bcl-xL	B-cell lymphoma-extra large
BNIP3	Bcl-2 and adenovirus E1B 19-kDa-interacting protein 3
BTG1	B-cell Translocation Gene 1
CAFs	cancer-associated fibroblasts
CC	colon cancer
CD	Crohn’s disease
COL10A1	collagen α-1(X) chain
DAMP	damage-associated molecular pattern
DNMT1	DNA Methyltransferase 1
DRAM2	damage regulated autophagy modulator 2
E2	estradiol
ECM	extracellular matrix
EMT	epithelial–mesenchymal transition
ER	estrogen receptor
ERE	estrogen response element
EVL	Enah/Vasp-like
FATS	fragile-site associated tumor suppressor
FOXO3	Forkhead box O3
GPR30	G protein-coupled receptor 30
HIF-1α	hypoxia-inducible factor 1-alpha
HL	hodgkin lymphoma
HMGB1	high mobility group box 1
IBD1	inflammatory bowel disease protein 1
ICI	immune checkpoint inhibitors
KRAS	kirsten rat sarcoma
LC	lung carcinoma
LC3	light chain 3
MAP	mitogen-activated protein
MAP1LC3B	microtubule-associated protein 1A/1B light chain 3B
mER	plama membrane estrogen receptor
miR	microRNA
mTOR	mammalian target of rapamycin
NF-κB	nuclear factor kappa B
NHL	non Hodgkin lymphoma
NK	natural killer
NO	nitric oxide
NOS3	nitric oxide synthase 3
NSCLC	non-small cell lung cancer
ODC	ornithine decarboxylase
PERK	PKR-like ER kinase
PI3K	phosphatidylinositol-3-kinase
PtdIns3K	phosphatidylinositol 3-kinase
PTEN	phosphatase and tensin homolog
Rheb	Ras homolog enriched in brain
ROS TAM	reactive oxygen species tumor associated macrophages
TME	tumor microenvironment
UC	ulcerative colitis
ULK	Unc-51-like autophagy activating kinase
XCI	X chromosome inactivation
